# Combating Child Undernutrition through Community Participation and Action: Reviving Ballabgarh Mixture at Rural Villages of Haryana, India

**DOI:** 10.4269/ajtmh.24-0649

**Published:** 2025-03-18

**Authors:** Ankit Chandra

**Affiliations:** Centre for Community Medicine, All India Institute of Medical Sciences, New Delhi, India

Ms. Kapuria, who was in her 90s, had a stooped posture and slowly walked toward me in the drawing room and sat on the sofa. In a slightly trembling voice and with enthusiasm, she shared the recipe for the “Ballabgarh Mixture,” a locally prepared nutritional supplement for undernourished children that had been pioneered by the Centre for Community Medicine at All India Institute of Medical Sciences, New Delhi, India. Like a child, I sat on her sofa and made notes as my memory may not be as acute as Ms. Kapuria’s. What impressed me the most was how, in moments, she calculated the caloric and protein content of the mixture on her fingers—362 kcal and 15.2 grams of protein per 100 grams. I was amazed by her cognitive abilities at her age. She mentioned that, because of her old age and having lost her teeth, she still prepares a similar mixture for her own nutritional needs, but she omits peanuts to reduce fat intake. She gave me a gap-toothed grin and asked me to wait while she slowly walked to her kitchen.

The path that led me to Ms. Kapuria began months before when I took charge as medical officer in charge of the Primary Health Centre in Chhainsa. During our performance review with the community health workers, the proportion of underweight children in our area caught my attention as it flickered on the Excel sheet displayed on the screen. The proportion of underweight children has remained substantial over the years. These data were not solely from immunization centers but also included new household data collected by our community health workers. These health workers expressed their frustration, stating that, although they are tasked with collecting data on malnutrition, it ultimately feels futile as no meaningful intervention is done. They pointed out that the nutritional support provided to children was insufficient and delivering such support does not fall under the purview of the health department.

Luck had it that I was able to join the dinner time discussion of the renowned Professor Emeritus Suresh Kumar Kapoor, at a primary healthcare continuing medical education program in Delhi. Over dinner, he spoke passionately about a remarkable initiative called the “Ballabgarh Mixture.” With animated gestures and a glimmer of nostalgia, he recalled that, when he was in charge of the child welfare center at Ballabgarh, he had witnessed the limitations of health education and medical treatment in addressing the complex social issue of undernutrition among children. Faced with this challenge, Dr. Kapoor and his team sought innovative solutions for nutritional rehabilitation. Because of the financial constraints, they reached out to affluent villages in the region, requesting donations of wheat and lentils. From these contributions, the “Ballabgarh Mixture” was born. Dr. Kapoor fondly credited Ms. Uma Kapuria, health educator, who used to prepare the mixture in a small grinder. This historical mixture was provided monthly to undernourished children at Ballabgarh, actively from 1985 to 1993, and they saw a marked improvement. Regrettably, this groundbreaking initiative was never formally documented. Listening to Dr. Kapoor was a eureka moment for me. It struck me that this approach could address the problem of underweight among children in Chhainsa, as I am facing the same problem. Excited to explore more, I reached out to senior health supervisors, and I was able to obtain Ms. Kapuria’s contact information. I arranged a meeting with her. On a crisp Sunday morning, I drove the hour to her home.

That is how I ended up sitting on the sofa of Ms. Kapuria brimming with anticipation. Ms. Uma Kapuria graciously shared the recipe for the mixture, which consisted of locally sourced ingredients: wheat, Bengal gram, green gram, and peanuts or sesame seeds in a ratio of 5:3:1:1. The mixture was formulated and modified over time, and importantly, it used ingredients donated by the village residents. These ingredients were roasted and ground to form a mixture, which was distributed in packets as a nutritional supplement for malnourished children.

This mixture can be mixed with milk or water to feed the child. By adding ghee and sugar, the mixture can be transformed into local sweets like panjeeri, ladoos, and halwa. When ground more fine, it can be used to make chapatis or parathas.

She and Dr. Kapoor would gather all the children visiting under five clinics at Ballabgarh to sit together and eat the Ballabgarh mixture as hot porridge. This strategy was particularly effective for children who often refused to eat when alone at home. By observing and eating alongside their peers, the children were more cooperative and willing to try the new food.

Ms. Kapuria smiled talking about the one time she made a lollipop out of it.

When Ms. Kapuria went to the kitchen, I waited on the sofa. As she walked slowly back from the kitchen, I noticed a small bowl in her hands. It was filled mixture for me to taste. It was delicious and far better than any protein or energy-dense supplement available in the market.

As I left Ms. Kapuria’s home, I felt inspired and determined to revive this historic mixture for the children. While driving back, I noticed the wheat ripening in the fields for harvest season. The next day, Mr. Bhagat Ram came to my office to request leave. I thought he might be involved in farming and could help in arranging the wheat. I was wrong, but when I shared my plan to revive the Ballabgarh mixture, and he became excited. Mr. Bhagat informed me that he had previously been involved with the Ballabgarh mixture initiative. He was familiar with several philanthropic and wealthy farmers in Chhainsa village who could potentially donate wheat. I asked him to list possible donors, and we set aside a holiday to meet them individually to discuss our plan. It was a resounding success. Given our goal of helping the village children, we managed to gather almost 200 kg of wheat, while a few donors even sold their wheat to buy peanuts, Bengal gram, and moong dal. Some of our staff members also contributed raw materials. Eventually, we had to pause the collection because of limited storage facilities.

I was delighted to see the enthusiasm of our team. Everyone wanted to be part of this new initiative. Our mess workers, gardeners, plumber, and sanitary workers took responsibility for washing, drying, and roasting all the ingredients. With our goodwill spreading, Mr. Bhagat brought in a community volunteer from Chhainsa village, who ground all the ingredients for free. The grinding machine was mounted on a large tractor trolley and had wheels at the bottom for easy transportation. During the grinding process, the machine got stuck because the peanuts made the mixture a bit oily.

I panicked and called Ms. Uma Kapuria for her advice. She suggested grinding the peanuts separately and mixing them later—a tip that saved the day.

In the end, we prepared about 100 kg of the Ballabgarh mixture and packaged it into 1 kg packets. Everything was locally procured and prepared. First, we tasted it ourselves (staff and health workers) as a sweet panjeeri. Then, we instructed our community health workers to explain the details and distribute this locally prepared mixture to the parents of undernourished children aged 1 to 5 years old who were willing to feed it to their children. After 15 days, we measured the children’s weight and gathered feedback. We also shared the recipe with mothers so they could prepare the mixture at home. Out of 73 children who received the mixture, follow-up was done for 53 children. Impressively, 33 children (58.9%) reported weight gain with a median increase of 300 grams. These results were encouraging and mirrored the positive outcomes that were reported by Dr. S.K. Kapoor.

After sharing these results with our team, they felt a sense of fulfillment for their efforts. However, I noticed that preparing the Ballabgarh Mixture was overburdening our workers and causing additional stress. Preparing it on such a large scale, keeping it protected from rodents and mites and ensuring safety levels was another concern.

We held meetings at the subcenters in the villages. These meetings were attended by Sarpanch (village head), village representatives, and mothers of young children. Together, we explored the causes and effects of undernutrition in children and brainstormed sustainable and effective solutions to tackle child undernutrition. All the suggestions were listed on chart paper, and each village resident participating in the meeting was given a small pebble to vote for their preferred solution.

This participatory approach allowed us to focus on the most popular option. Based on the voting results, the majority of people voted for improving nutritional services and support through existing Anganwadi centers (government-owned childcare and nutrition centers), as well as enhancing the nutritional education of mothers. For areas where Anganwadi performance was inadequate or for children from marginal families needing additional support, participants suggested that the Ballabgarh mixture be prepared at the village level by volunteers and distributed locally.

With the help of community health workers, we were able to identify volunteers in each village who were ready to prepare and distribute the Ballabgarh mixture monthly to children who are severely undernourished or belong to vulnerable families. These volunteers were primarily village Sarpanchs, but in some villages, community health workers and village residents took the lead. We taught the Ballabgarh mixture recipes to the volunteers and mothers, and provided counseling and educational pamphlets regarding the nutritional care of children. Simultaneously, our community health workers collaborated with Anganwadis to identify undernourished children in the community. Over the next few months, we continued this effort, and in our monthly reports, we saw a decline in the prevalence of underweight children.

This story serves as a testament to the enduring impact of innovative, collaborative, and community-driven health interventions to combat child malnutrition sustainably. I extend my heartfelt thanks to my entire team, Dr. SK Kapoor and Ms. Uma Kapuria for their guidance.

